# Updates in central nervous system malaria: literature review and considerations

**DOI:** 10.1097/QCO.0000000000000829

**Published:** 2022-04-28

**Authors:** Andrea Marino, Dalida Angela Bivona, Paolo Bonacci

**Affiliations:** a Department of Biomedical and Biotechnological Sciences; b Department of Clinical and Experimental Medicine, Unit of Infectious Diseases, ARNAS Garibaldi Hospital, University of Catania, Catania, Italy

**Keywords:** cerebral malaria, malaria, *Plasmodium falciparum*, severe malaria

## Abstract

**Purpose of review:**

Cerebral malaria (CM) represents one of the most common and severe complications of *Plasmodium falciparum* infection, leading to high morbidity and mortality along with challenging sequelae, especially in children.

**Recent findings:**

Although CM pathogenesis remains unclear due to the few studies made and the difficulty to analyze affected patients, there are valid theories involving *P. falciparum* endothelium interactions, and clinical manifestations have been better investigated and differentiated between adults and children.

**Summary:**

At the time of writing, diagnostic management is based on fast severe malaria identification by blood smear (thin and thick). However, newer techniques involving molecular testing (such as PCR or LAMP) and biomarkers identification are now available. It is also important to check patients’ cerebral functions. As regards therapeutic management, although we could rely on several options, artesunate represents the gold standard treatment. Cerebral complications such as seizures and coma need to be managed as well.

## INTRODUCTION

Malaria is an infectious disease caused by a protozoan, a parasitic microorganism of the genus *Plasmodium*, which is transmitted to humans through the bite of mosquitoes of the genus Anopheles. Infected mosquitoes are called “malaria vectors” and the disease is defined as a vector-borne and zoonotic infection [1].

Malaria is a major worldwide health issue representing the primary cause of morbidity and mortality in many countries. Even though not all cases are fatal, malaria still kills over 430,000 people (predominantly children) every year in endemic areas such as Sub-Saharan Africa [2].

New cases of disease in nonendemic areas are mainly linked to tourists or immigrants who return or come from countries where malaria is endemic.

Among Plasmodium spp., *Plasmodium falciparum* is responsible for malignant or tertian malaria. In particular, cerebral malaria (CM) is one of the most common and fatal complications associated with *P. falciparum* infection [3].

It is estimated that around 1% of children infected with *P. falciparum* develop a more severe form of malaria which may eventually lead to cerebral complications, including CM [4]. 

**Box 1:**
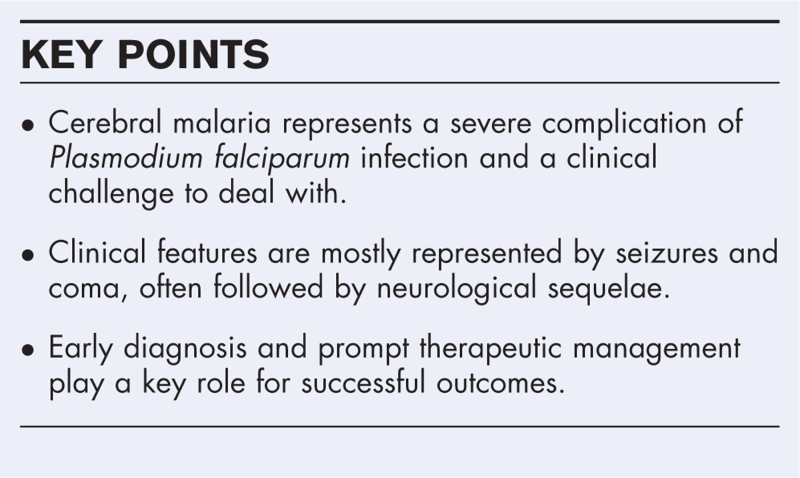
no caption available

## PATHOGENESIS: THEORIES AND HYPOTHESIS

CM could be defined as the most common nontraumatic encephalopathy in the world. The pathogenesis often differs between adults and children and the spectrum of malaria manifestation can be very broad, ranging from asymptomatic infections to life-threatening syndromes [5]. The severe form of the disease is characterized mainly by coma and the presence of the asexual form of the parasite [6].

Furthermore, the infection process depends on several factors: human genetics, malaria parasite genetics, nutritional status, and intercurrent infections [7]. Pathogenesis of CM includes mechanical obstruction of brain microvasculature by sequestered parasitized red blood cells, inflammation, hemostatic dysfunction, excessive parasite-derived lactate, and oxidative stress [8].

During intra-erythrocytic maturation, *P. falciparum* exports proteins that significantly alter the host cell membrane, allowing infected RBCs (iRBCs) to bind to endothelial cells in blood capillaries and postcapillary venules of the brain [6]. Members of the *P. falciparum* erythrocyte membrane protein 1 family mediate iRBC adhesion to endothelial cells by binding to receptors such as cluster of differentiation 36 (CD36), intercellular adhesion molecule 1 [9], and endothelial protein C receptor (EPCR) [10]. In addition, Ortolan *et al.* raise the possibility of a brain-gut-kidney binding axis contributing to multiorgan complications in severe malaria involving Group A EPCR-binding subsets [11^▪▪^].

This mechanism is involved in the development of cerebral hypoxia with particular reference to the local reduction of oxygen consumption in the brain as a consequence of vascular obstruction [12], to cytokine-driven changes in glucose metabolism, and to cytopathic hypoxia [7].

To develop appropriate neuroprotective mechanisms, it is necessary to understand how an intravascular parasite causes such brain injury [13].

In a recent study [14^▪▪^] it has been hypothesized that NOX2 overexpression may result in loss of hippocampal neuronal density and CA1 neurons dendritic spines affecting spatial working and reference memory during experimental CM.

Microvascular congestion appears to cause severe endothelial damage, resulting in vessel wall disruption, myelin and axonal damage, and a breakdown of the blood-brain barrier (BBB) [15,16].

This aspect should be especially noted in children, which could be due to the ongoing brain development in young children making the BBB more susceptible [17].

Schappo *et al.*[18^▪▪^]. examined the genetic diversity, antigenicity, and adhesiveness of *Plasmodium vivax* VIR-E protein since its hypothesized pathogenetic role.

## CLINICAL MANIFESTATIONS

### Manifestation between children and adults

Arguably due to age-related immunity states, clinical CM shows several differences between children and adults in terms of frequency, severity, and neurological sequelae [19,20].

In broad terms, coma, seizures, and neurological deficits are both more frequent and severe in children than adults, as well as systemic complications and retinopathy [20].

As regards neurological sequelae, children seem more involved in cognitive impairment and epilepsy development, whereas sequelae in adults are less common [20].

Every aspect of those clinical diversities is explained in the paragraphs below.

### Acute manifestations

CM is a syndrome comparable to a diffuse encephalopathy, primarily characterized by the presence of seizures, loss of consciousness (up to coma), or both [19].

In endemic areas, CM typically interests children and exhibits a coma (lasting 1 or 2 days), which follows seizures that are frequent and mainly partial motor, showing a high prevalence of status epilepticus development; in adults, it is more often characterized by multiorgan dysfunction following generalized symptoms such as malaise, fever, joints and body aches, and headache up to delirium and coma [3,4,21,22].

Although coma recover time in adults is longer than in children, adults seem to be less susceptible to seizures and status epilepticus [5–7].

In addition, adults experience less persistent neurological sequelae compared to children, which are mainly characterized by focal neurological deficit, upper motoneuron lesion, disconjugate gaze, decorticate/decerebrate rigidity, cerebellar ataxia, hemiplegia, extra pyramidal rigidity, and psychosis; however, the development of these sequelae in adults correlates with higher chances of poor prognosis outcome [23].

CM systemic complications, involving both children and adults, are mainly represented by hyponatremia, severe anemia, hypoglycemia, and metabolic acidosis causing respiratory distress [24,25]. Moreover, although some authors [26] reported a high frequency of high intracranial pressure in patients with severe malaria, others [26] demonstrated that mannitol did not ameliorate clinical outcomes.

### Neurological sequelae

Neurological deficits as long-term sequelae, frequent among children, include epilepsy, paresis, ataxia, hemiplegia, persistent cortical blindness, deafness, cognitive impairment, disruptive behavior, and language deficits [26–28].

These manifestations may resolve from 6 months to 9 years after discharge [27]; conversely, other studies [29,30] evinced that the neurological deficits, especially cognitive impairment, may deteriorate over time.

Furthermore, long-term cognitive impairment may correlate with severe malarial anemia, whereas attention deficits along with memory impairment are caused by cerebral complications, such as multiple seizures, severe hypoglycemia, prolonged coma, and intracranial hypertension [31].

Abnormalities regarding temporal, motor, and cognitive areas appear first, followed by behavioral dysfunction and eventually epilepsy [32]. Language disorders were reported in almost 12% of CM survivor children, specifically in phonology, lexical semantics, receptive vocabulary, and pragmatics [32].

### Malarial retinopathy

**FIGURE 1:**
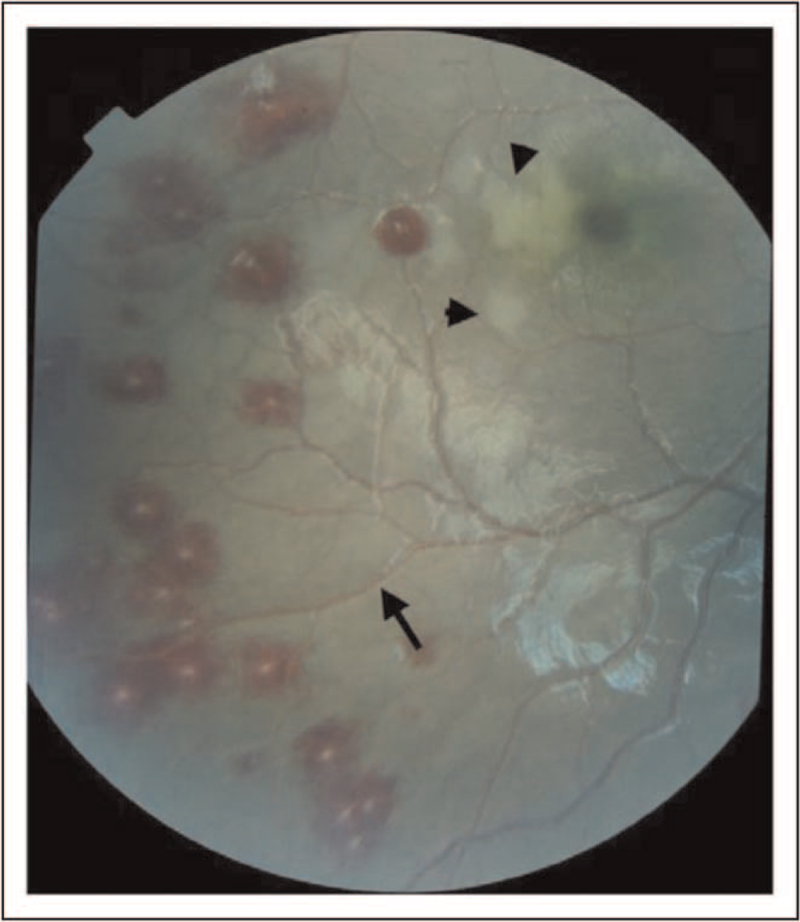
Fundus examination of a patient with malarial retinopathy. Reproduced from White VA, Lewallen S, Beare NAV, Molyneux ME, Taylor TE (2009) Retinal Pathology of Pediatric Cerebral Malaria in Malawi. PLoS ONE 4(1): e4317. https://doi.org/10.1371/journal.pone.0004317.

Moreover, although less common in adults, retinopathy represents a frequent complication of CM (Fig. 1), characterized by four main features: retinal hemorrhages, retinal vessel discoloration (to pink-orange or white), retinal whitening (involving macula and sparing central fovea along with fundus peripheral whitening), and papilledema resolving within four weeks after discharge without ocular sequelae [10,13,21]. Interestingly, the presence of papilledema without retinopathy should suggest a nonmalarial cause of cerebral damage with increased intracranial pressure [33].

Retinal findings in CM are caused by RBC sequestration phenomena and microcirculatory impairment leading to occlusion of retinal vessels and hypoxia [33,34].

Malaria retinopathy (MR) represents both a valuable diagnostic marker, as it helps to distinguish between malaria encephalopathy and other forms of central nervous system involvement thanks to the fact that retinal whitening and vessel changes are specific to malaria, and a prognostic tool, as there is a correlation between both the number of retinal and cerebral hemorrhages [33] and severity of malaria-specific retinopathy and neurocognitive outcomes [34].

## DIAGNOSTIC MANAGEMENT

Worldwide, the majority of deaths caused by malaria are provoked by delayed diagnosis and deferred treatment [35]. Every febrile patient from an endemic area should be tested for malaria. Moreover, since CM is a *P. falciparum* infection feature, it is critical not only to diagnose the infection, but also to be able to distinguish the different plasmodium species in time. Characteristics of commonly used diagnostic methods are described in Table 1[36].

**Table 1:** Characteristics of most used diagnostic methods for diagnosis of malaria

Diagnostic Method	Specificity	Sensitivity	Advantages	Disadvantages
Microscopy	100%, gold standard	50–500 parasites/μL	Very specific and accurate Provides both quantitative (parasite density/parasitemia) and qualitative (Plasmodium species) dataUseful to diagnose the stage of malaria infectionCould be used to monitor antimalarial therapy	Requires highly trained personnelRequires reliable electricity supplyLow sensitivity (depending on laboratories)
Rapid diagnostic test (RDT)	About 90% (higher for High Sensitivity Tests)	2.6–14.6 ng/mL of *P. falciparum* histidine-rich protein 2 (PfHRP-2).	Provides results very quicklyInexpensiveEasy to use with no/little training	Cannot differentiate between past and present infectionsUnable to quantify parasite densityMutation in the gene encoding the antigens could affect the results
Immunology-based	90–95%	100 parasites/μL	Provide information on malaria epidemiological surveillance	Cannot differentiate between past and present infections
Molecular-based [PCR/LAMP]	Near to 100%	1–5 parasites/μL	Highly sensitive and specificDistinguish between different Plasmodium species	Requires highly trained personnelVery expensive equipmentIt is time-consuming

PCR, polymerase chain reaction; LAMP, loop-mediated isothermal amplification.

Although CM symptoms usually occur within two weeks after the infection, in children and immunosuppressed subjects they may develop in 12 h.

According to the WHO, CM should be suspected whenever a patient can not localize painful stimulus, presents *P. falciparum* parasitemia, and does not present other causes of encephalopathy [20].

Although a patient's epidemiological and clinical history could help suspect the disease, conclusive diagnosis of malaria requires parasites direct observation in Wright- Giemsa blood smears (thick and thin). Since it is not simple to identify Plasmodia as its forms may vary during its life cycle, leading to several false positive and negative being reported, only skilled staff should perform the test.

A recent study [37^▪^] revealed that hemoglobin levels are lower in patients infected with *P. falciparum* than in those infected with other Plasmodia, whereas other markers such as bilirubin, AST, and ALT are similar for all Plasmodia.

Royo *et al.*[38]. showed that the percentage of nonclassical monocytes is higher in the uncomplicated malaria group than in those with severe/CM.

New diagnostic methods recently introduced [37^▪^,^38^–^41^] include rapid antigen-capture dipstick test, which could differentiate between *P. falciparum* and nonfalciparum malaria, and the use of a fluorescent stain to detect the plasmodia; both are fast, highly effective, and easy to perform, even without specific expertise [42^▪^,^43^,^44^,45^▪^].

Current clinical criteria for CM diagnosis misclassifies 25% of patients; however, when adding MR, the diagnostic yield rises to 95%.

As a matter of fact, Joshi *et al.*[43]. developed an automated software to detect MR lesions early, since ocular fundus examination needs expensive resources and expertise that are not available in several malaria-endemic areas.

Other research groups [44,46] developed several clinical algorithms to diagnose CM more accurately and more efficiently.

The most recent method for identification and/or distinction of CM from other forms is represented by biomarkers identification. Omar *et al.*[47^▪▪^] discovered up to 60 gene signatures that may discriminate between severe and CM, and that may be used as blood a diagnostic tools in endemic countries to determine malaria severity.

Furthermore, it is essential to role out potential coinfections, especially viral coinfections, such as HCV, HBV, and HIV, which could deteriorate malarial clinical trends, as well as bacterial superinfections [48–51].

## TREATMENT CONSIDERATIONS

Severe/CM is almost always caused by *P. falciparum*; *P. vivax* and *Plasmodium knowlesi* can less commonly cause severe malaria, but the treatment approach is the same. Parenteral artesunate represents the first-line therapy. It does not need any modification as regards kidneys and liver function; however, it is important to check for drug-drug interactions since artesunate is metabolized by cytochrome P450. The single dose of iv artesunate is 2.4 mg/kg at 0, 12, and 24 h [52–54].

As soon as the patient may tolerate oral therapy and after at least 3 intravenous artesunate administrations, it is recommended to switch to one of the two approved oral formulations (artemether/lumefantrine or dihydroartemisinin /piperaquine), starting at least four hours after the last iv administration, for three days of treatment [52].

On the contrary, if the patient is not able to assume an oral formulation, it is suggested to continue with artesunate iv adding clindamycin; artesunate iv should not be given for more than 7 days in total. IV artesunate has been associated with delayed hemolysis occurring a mean of 15 days post therapy, especially in nonimmune travelers [54].

Iv quinine dihydrochloride represents the second treatment of choice since it is significantly inferior to iv artesunate which has shown a remarkable mortality reduction cerebral malaria in 2 definitive RCTs [54]. For those reasons, WHO recommends artesunate iv as the gold standard treatment for severe and CM [52].

See Table 2 for recommended adult regimens in patients with malaria.

**Table 2:** Treatment options for malaria infection

		Therapeutic options	Recommended adult regimens
Nonsevere *Plasmodium falciparum* malaria	Acquired in areas with chloroquine resistance (or resistance unknown)	Artemether-lumefantrine (1 tab: 20 mg artemether and 120 mg lumefantrine)	4 tabs po per doseThree-day course:Day 1: Initial dose and second dose 8 h laterDays 2 and 3: 1 dose BID
		Dihydroartemisinin-piperaquine (1 tab: 320 mg piperaquine and 40 mg dihydroartemisinin)	4 tabs po QD × 3 days
		Atovaquone-proguanil (1 tab: 250 mg atovaquone and 100 mg proguanil)	4 tabs po QD × 3 days
		Quinine sulfate + doxycycline or clindamycin	Quinine sulfate: 542 mg base (650 mg salt) po TID × 3 or 7 days 7 Doxycycline: 100 mg po BID × 7 daysTetracycline: 250 mg po QID × 7 daysClindamycin: 20 mg/kg/day po divided TID × 7 days
		Mefloquine	Dose 1:684 mg base (750 mg salt) poDose 2 at 6–12 h: 456 mg base (500 mg salt) po
	Acquired in areas without chloroquine resistance	Artemether-lumefantrine	See above
		Dihydroartemisinin-piperaquine	See above
		chloroquine phosphate	Dose 1: 600 mg base (1,000 mg salt) poDoses 2 to 4 (3 additional doses) at 6, 24 and 48 h: 300 mg base (500 mg salt) po per dose
		Hydroxychloroquine	Dose 1: 620 mg base (800 mg salt) poDoses 2 to 4 (3 additional doses) at 6, 24 and 48 h: 310 mg base (400 mg salt) po per dose
Severe *Plasmodium falciparum* malaria (Drug susceptibility not relevant for acute treatment of severe malaria)		IV artesunate (1 dose = 2.4 mg/kg)If IV artesunate not readily available, give oral antimalarials while obtaining IV artesunate (see above for dosing): -Artemether-lumefantrine (preferred); or-Atovaquone-proguanil; or-Quinine sulfate; or-Mefloquine (only if no other options available)	IV doses (3 in total) at 0, 12 and 24 hPLUSReassess parasite density at least 4 h after the third dose.Parasite density ≤1% and patient able to tolerate oral medications: Give a complete follow-on oral regimen.Options include (See above for dosing): Artemether-lumefantrine (preferred), or Atovaquone-proguanil, or Quinine plus doxycycline or, in children < 8 years old and pregnant women, clindamycin, or Mefloquine (only if no other options available)Parasite density >1%: Continue IV artesunate, same dose, QD up to 6 more days until parasite density ≤1%. When parasite density ≤1%, give complete follow-on oral regimen as listed above (See above for dosing).Parasite density ≤1% but patient unable to take oral medication: Continue IV artesunate, same dose, QD up to 6 more days until patient able to take oral therapy.

BID, twice a day; h, hour(s); IV, intravenous; po, by mouth; QD, once a day; QID, four times a day; tab(s), tablet(s); TID, three times a day. Parasite density should be repeated every 12–24 h until negative. As regards pediatric and pregnant women regimens and dosing see CDC recommendations and WHO guidelines.

Although double therapy superiority has not been demonstrated, quinine/artesunate iv association is recommended to treat severe malaria in countries were high incidence of artesunate-resistant malaria is documented (e.g. Cambodia, Laos, Thailand, Vietnam) [45^▪^,^55^,^56^]. Furthermore, it is strongly advised to monitor patients very cautiously, better if in an ICU. Continuous electroencephalogram monitoring may detect subclinical crisis; a frequent GCS evaluation should be considered in order to assess potential mental deterioration, and brain CT scan could rule out other causes of altered consciousness (cerebral hemorrhage or cerebral herniation) [57]. Convulsive crises should be treated with antiepileptic drugs (diazepam or phenytoin) and status epilepticus could be tackled with deep sedation (propofol, BDZ, and barbituric drugs).

Dexamethasone and mannitol did not show any beneficial effect in edema reduction, whereas corticosteroids utilization has been associated with increased risk of bleeding and seizures along with prolonged coma recovery time. Even mannitol iv administration correlates with high risk of prolonged coma. It should be discouraged the prophylactic use of antiepileptic drugs due to their association with a worse outcome.

Some studies showed that pentoxifyilline, a phosphodiesterase inhibitor, leads to a mortality reduction as well as decreasing coma recovery time by reducing TNF levels; however, other studies did not confirm any beneficial effects when compared to placebo, whereas one study reported a higher mortality rate [58].

## CONCLUSION

An early diagnosis and medical intervention are crucial in the fight against CM, as they prevent the parasite from maturing inside the erythrocyte and causing catastrophic damage, specifically cerebral anoxia. Furthermore, it appears that, when infected with malaria, the host initiates a variety of protective tissue responses that are probably overwhelmed by severe malaria; in particular, CM could be the result of an hyperstimulation of the immune system as it tries to fight the infection. Enhancing the host's defences could be a way to slow the spread of severe malaria until antimalarial medication, such as artemisinin derivatives, can eradicate the parasite. Another unifying goal must be the development of new antimalarial medications or vaccines capable of protecting against the disease's severe forms such as CM itself.

### Uncited references

[28,40,41].

## Acknowledgements

*We thank Dr Pietro Leanza for his kind English revision.*

### Financial support and sponsorship

*None.*

### Conflicts of interest

*There are no conflicts of interest.*

## REFERENCES AND RECOMMENDED READING

Papers of particular interest, published within the annual period of review, have been highlighted as:

▪ of special interest
▪▪ of outstanding interest
